# Resilience: Myanmar students’ experiences of overcoming eLearning challenges during COVID 19 and political instability

**DOI:** 10.1007/s12564-022-09781-6

**Published:** 2022-08-15

**Authors:** Steve Gomersall, Alan Floyd

**Affiliations:** 1 Brighter Futures Pathways, 70 Hloung Daw Mu Road, Tawan Tar Village, Hpa-an, Kayin State Myanmar; 2 Institute of Education, University of Reading, London Road Campus, 4 Redlands Road, Reading, RG1 5EX UK

**Keywords:** Resilience, COVID-19, Myanmar, eLearning

## Abstract

In 2020, COVID-19 forced global education online. Especially in developing countries, this change presented a challenge for those with limited access to devices and reliable electricity and Internet. Whilst some students struggled to adapt, others thrived with eLearning. This paper explores the experiences of one group of students who succeeded in overcoming not only the restrictions of COVID-19, but also significant political instability. The group has a mix of 1st year and 2nd year students in a pre-university program. The 2nd-year students in particular are noteworthy as despite the adversity faced, they have now successfully transitioned to university studies. Drawing on Bronfenbrenner’s (The ecology of human development: experiments by nature and design, Harvard University Press, Cambridge, 1979) socio-ecological framework, the data from twelve semi-structured interviews was analysed and inductively coded. The findings outline how personal goal orientation, coping mechanisms acquired in previous adverse situations, and a desire to serve their communities combine with external influences from families and communities and basic need fulfilment to enable the students to persevere and thrive. The implication is that as students draw on previous experiences, these five core concepts should be incorporated into general education to build the students’ capacity for resilience.

**Supplementary Information:**

The online version contains supplementary material available at 10.1007/s12564-022-09781-6.

The 2020–2021 academic year was an *annus horribilis* for education around the globe. With widespread school closures, pupils and parents struggled to adapt to online learning, especially in areas of high deprivation and limited access to appropriate equipment and Wi-Fi connections. Moreover, many teachers and professors suddenly had to transfer their classes online and grapple with the new technological demands despite insufficient knowledge and training of eLearning strategies. Researchers in the West have been proactive in examining how schools and universities experienced and managed this process (see, for example, https://www.open.ac.uk/projects/leading-online-learning/ in the UK and Appolloni et al., 2021 in Italy). However, less is known about the situation in developing nations, although one recent study noted a disparity between urban and rural areas in Ethiopia, India, Peru, and Vietnam (Hossain, 2021).

In Myanmar, the ruling National League for Democracy closed schools for most of the 2020–2021 academic year, yet few resources were transferred online. Those students who continued their studies did so via private tutors, learning centres and informal networks. Following a disputed election in November 2020, the overturning of the results and widespread political instability,^1^ students seeking to continue their education faced even greater adversities than their peers in similar developing nations. Nevertheless, within this context, some students thrived and not only completed their studies, but also gained admission to international universities. This paper, we will seek to explore the factors that led one such group of students, from economically deprived backgrounds, to demonstrate resilience in the face of overwhelming adversity.

Research shows that resilience is a multi-faceted construct, with links to the individual, local environment and wider community and culture (Noltemeyer & Bush, 2013; Ungar, 2011). To provide new theoretical insights into this complex area, this study will utilise the socio-ecological framework of Bronfenbrenner (1979) to look at the interconnected domains within which an individual simultaneously exists, to identify contributing factors to the development and deployment of resilience within an under-researched population group. Thus, the article provides an original and significant contribution to our knowledge in this field.

The goal of the study is to investigate the following research questions:

RQ1: How did previous life experiences affect the students’ resilience?
RQ2: What internal/individual factors assisted students to be resilient in their studies?
RQ3: What external/community factors assisted students to be resilient in their studies?

Following this introduction, the paper is organised into six subsequent sections: background information about the local context; the conceptual framework; description of the methodology; presentation of the findings; analysis of the data; and discussion of the conclusions.

## Local context

When Myanmar gained independence from Britain in 1947, its education system was one of the most developed in South East Asia; however, due to decades of political instability, chronic underfunding, and a lack of modernisation, the current system is crumbling (Lall, 2008). Despite the National Educational Strategic Plan, published in 2015, by 2019 only 2.0% of GDP was spent on education, compared to a world average of 3.7% and an average of 3.1% in countries classified as “fragile and conflict affected” (UNHCR, 2021). The problem is exacerbated in both rural and ethnic–minority areas; as high schools were generally opened in towns and cities, rural families either had to pay for expensive dormitories or organise transportation, which was particularly difficult in monsoon seasons. Furthermore, many ethnic armed organisations have had long running on–off civil wars with the Myanmar government. This led to many people from ethnic minorities fleeing to refugee camps along the Thai–Myanmar border or migrating to Thailand for employment opportunities (Eberle & Holliday, 2011).

In response, numerous informal migrant learning centres opened in Thai border towns, and refugee committees formed schools in refugee camps. As none of these centres are recognised by either the Thai or Myanmar governments, the graduates leave school with unaccredited certificates and diplomas (World Education, 2014). In general, students in these centres face challenges throughout their educational journey. Students must complete each grade sequentially, and thus there is a wide age range in each class (Nawarat, 2018). The lack of accreditation particularly impacts on the possible progression to university for students outside mainstream education. After completing Grade 12, those wishing to pursue tertiary education often need to enrol in programs preparing students for the American General Education Development high school equivalency examination, or else apply to special university courses run locally such as by the Australian Catholic University, or online to low cost courses such as the University of the People (Lwin et al., 2022).

Since 2007, several programmes have opened that help students to gain a recognised high school diploma and access scholarships to support their further education. This case study is based on one such training programme run by the first author, in Hpa-an, the capital of Kayin State. Students in this pre-university training programme are all over the age of 18, and study for 1–2 years before applying to university. As this programme targets students from the poorest backgrounds, tuition and accommodation in the boarding house are free, with students only paying for their uniforms. For ethical reasons, it will be referred to as the Maung Kyaw Programme. The students whom the first author has worked with, through his role as the founder and director of Maung Kyaw Programme, often have strong academic skills, fluency in three or more languages, and a desire to continue to higher education to improve their own lives and develop their communities. Since the focus of Maung Kyaw Programme moved to university preparation in 2015, over 90% of the enrolled students have passed their GED examinations and 73% progressed to university.

In 2020, with coronavirus spreading, most countries began to enter lockdowns which necessitated the closure of schools and businesses, and a flexible approach to management and problem solving (see, for example, Binci et al., 2020; Rajak et al., 2022). Many governments responded to this crisis by asking schools to transfer teaching online; however, in Myanmar, where the school year commences in June, institutions were asked to remain closed. In response, Maung Kyaw Programme began to deliver online instruction via content hosted on Facebook. When high schools were allowed to reopen in August, the students made their way to school. Three weeks later, schools were asked to close again as COVID-19 was spreading. In Maung Kyaw Programme, those able to safely return home did so, and those unable due to travel restrictions, remained in the dormitory. All reverted to online lessons. However, as identified in comments by the former State Counsellor, Daw Aung San Suu Kyi, online learning was not widely suitable for students in Myanmar, where there is a lack of infrastructure, compared to developed nations (Mon, 2020). This complex educational and social context provides the backdrop for this study.

## Theoretical framework: the concept of resilience

### Defining resilience

At the outset, it is essential to define what we mean by the term ‘resilience’. Definitions of resilience vary from the idea of robustness and minimal disruption (Limnios et al., 2014) to the concept of thriving and positive adaptation (Gillham et al., 2013). In a systematic review of interdisciplinary research, Windle (2011) identified three essential aspects of resistance: adversity, utilisation of resources, and adaptation and summarised that:

Resilience is the process of effectively negotiating, adapting to, or managing significant sources of stress or trauma. Assets and resources within the individual, their life and environment facilitate this capacity for adaptation and ‘bouncing back’ in the face of adversity. (p.163)

As well as being based on a comprehensive review of research, this definition encapsulates the key concepts which we will explore, and therefore, this is the definition used throughout this article.

Viewing resilience as the response to negative experiences, Schwarze and Wosnitza (2018) outlined that there is a range of potential adverse causes from the ‘micro-stressors’ of daily life to significant events causing chronic and emotional change. In this paper, we will focus on the impact of COVID-19 restrictions and nationwide political instability on a group of students in Myanmar, both long-term, chronic antecedents.

### Socio-ecological model of resilience

In 1979 Bronfenbrenner outlined his ecological approach to studying childhood development, which viewed the individual as simultaneously occupying multiple positions in society. The four key positions were termed the microsystem, mesosystem, exosystem, and macrosystem, which Bronfenbrenner described as being akin to “a set of Russian dolls” (Bronfenbrenner, 1979, p. 3). The microsystem referred to the individual’s identity and interpersonal relationships in a local setting. This expanded at the mesosystem to consider the interactions between the different settings which the individual inhabited, such as between the actors in the family and school setting. The exosystem referred to the wider contexts which did not impact upon the individual directly, but would influence the mesosystem, and the macrosystem referred to the overall culture.

Drawing on Bronfenbrenner, research into resilience formation has utilised the socio-ecological framework to consider interactions between the various environments that a person experiences, from the individual to societal (Ungar, 2011). This approach has the advantage of acknowledging that resilience is a multi-dimensional construct, developed through interactions with people and the local context, and thus avoids reducing discussion to a narrow set of factors (Gu, 2018). This is particularly pertinent for this study, as COVID-19 and associated restrictions had the potential to simultaneously affect multiple domains by increasing psychological disorders, disrupting family relationships, adversely impacting upon finances, and limiting people’s freedom (Prime et al., 2020). By utilising the ecological lens, resilience has been shown to be an ongoing process, which develops over time as the individual is nurtured and learns from their environment and experiences (Gu, 2018). This process involves individuals’ internal self-awareness, interactions with supportive others, and external community resources, such as access to food and education (Ungar, 2013).

The factors highlighted in the socio-ecological model have significant commonality with the needs outlined in the hierarchical model of motivation proposed by Maslow (1943) and we thus infer that being motivated, with needs fulfilled, is a necessary element of resilience. There has been extensive research into online learning utilizing the lens of Maslow’s hierarchy of needs; however, much of this is western-centric and ignores the digital poverty in developing nations. For example, Milheim (2012) commented that the physiological needs such as a computer, high-speed Internet and software are the easiest to fulfil. One of the original aspects of our study is the application of existing theoretical frameworks to an under-researched context.

### Utilization of resources

As identified by the socio-ecological model, individuals live within a complex set of relationships and settings and are influenced by a variety of societal actors. Noltemeyer and Bush (2013) produced a synthesis of international research into resilience which grouped factors leading to positive adaptation into four key areas: the individual, family, school opportunities, and the community, all of which are incorporated in the levels identified in the socio-ecological approach. These same four domains were identified by Henderson et al. (2016), who researched potential interventions to promote resiliency.

At the individual level, Werner (2000) found that young adults (17–18 years) were more likely to be successful in overcoming adverse situations if they had a strong internal locus of control and were well-liked by their friends and family. Linked to this is self-esteem, which can produce a more positive perception of the social support available and thus increase resilience (Sevil-Gülen & Demir, 2021). In addition, Hemson (2019) identified that those students succeeding were problem solvers, who found ways to continue their studies, despite financial problems and a lack of quality education. However, whilst Wills and Hofmeyr (2019) also note the correlation between resilience and perseverance, they argue it is not possible to determine causality without further qualitative studies.

Beyond intelligence and socio-emotional skills, several studies in a range of locations have identified the desire to achieve goals as being common amongst those who persevere in their education, as this would lead to improved lives and the capacity to help others (Dass-Brailsford, 2005; Keijzer et al., 2021; Motsa & Morojele, 2017; Rogers & Anderson, 2019). The participants in Motsa and Morojele (2017) articulated that educational attainment was the only way which they could attain a better future. Similarly, Smokowski et al. (1999) found that resilience was an ongoing process typified by “perseverance, determination, belief in a better future, and the maintenance of personal dreams and goals” (p. 435). Dass-Brailsford (2005) also identified the presence of a form of spirituality, as shaping the students’ direction and character formation.

Turning to the family, research has shown parents can have a strong reinforcing effect on their children’s educational attainment. Williams and Bryan (2013) found that the most important factor for the success of African–American children from single-parent families was the impact of parenting practices, such as praise for achievement and discipline for poor grades. Furthermore, siblings can have a significant impact, both offering emotional support, and sacrificing their own educational aspirations to financially support another sibling’s education (Rogers & Anderson, 2019).

Within the school environment, Sandoval-Hernándex and Bialowski (2016) found a strong correlation between students valuing their education and having supportive teachers who believed in their academic success and resilience, and in turn Ozeren et al., (2020) note a similar dynamic between supportive headteachers and the efficacy of teaching staff. Likewise, Mampana and Bouwer (2011) identified a correlation between the school’s emphasis on academic attainment and the individual student’s achievement orientation. Significantly, both Sandoval-Hernándex and Bialowski (2016) and Mampana and Bouwer (2011) were conducted amongst children of low socio-economic backgrounds, and thus demonstrated how resilience can overcome the disadvantage of economic status. Dass-Brailsford (2005) and Williams and Bryan (2013) equally concluded that teachers or other school staff had a positive influence on the students’ resilience, with Williams and Bryan (2013) finding that all the participants in their study had a close relationship with at least one staff member who not only offered academic guidance but also emotional support. Arnold and Doctoroff (2003) summarise that a positive relationship with an adult, whether parent or teacher, can serve as a protective factor in the resilience process, giving the child a role model, direction, and resources.

At the community level, community-centred interventions can assist youth to thrive (Henderson et al., 2016). Rogers and Anderson (2019) demonstrate societal support can take numerous forms including tangible financial aid or transportation, emotional support, and advice on how to access further studies. Moreover, in contexts, where there is armed conflict, the school setting has been shown to be viewed as a safe location, with girls who enrol in higher education experiencing lower levels of violence (Landis et al., 2018). This is particularly pertinent for our study as we are examining the experience of students facing not just COVID-19 restrictions, but also political instability in a country which has experienced ongoing civil wars for a protracted period of time.

Finally, it is important to note that not all of these factors need to be present for resilience to form. For example, in some less-developed regions community members may not value education and discourage students, but with other support factors in place, this can be overcome (Grace & Eng, 2020). The research questions guiding this paper explore how students drew on various strands of support and resources to become resilient.

## Methodology

To address the study’s research questions, this study adopted a relativist ontological approach informed by social constructivism. The goal was to understand the process of resilience formation in the target population. It is accepted that every context will have unique features and thus it is not the aim to generalise the findings but to offer rich insights into a complex social situation which may help others in similar contexts (Scotland, 2012). The credibility of the findings is dependent upon the transparency and accuracy of the process, and the sufficiency of the data to enable researchers and practitioners to judge the extent that the conclusions are transferable to their own settings (Nowell et al., 2017). Thus, this methodology section contains an auditable trail of the decisions we have taken.

### Sample

A case study approach was selected which used the insider position of the first author to purposively sample the students in his institute. Whilst utilisation of the insider has the potential to introduce bias, numerous studies have been conducted exemplifying the value this has for educational research (cf. Brydon-Miller & Maguire, 2008; Casey & Dyson, 2009).

At the start of the academic year, there were a total of 33 students across two classes. Due to COVID-19, students were given the option to return home and study online. Ten of the 33 enrolled students took this option, with the remaining 23 requesting to remain in the dormitory due to local travel restrictions. A safe space was given for the students to remain, although they also studied online for the whole year. Online lessons were generally delivered asynchronously, with instruction delivered through pre-recorded videos to enable students to study when they had a strong signal and free time. Of the original 33 students onsite in June 2020, 19 have progressed to university and five dropped out of their studies.

Purposive sampling (Cohen et al., 2017) was used for the selection of participants with only those who had successfully completed the academic year being invited to participate. During the year, five girls gained admission to university and so were removed from the potential pool of 33 participants. Of the remaining students, five (15%) dropped out due to the pandemic, leaving 24 potential participants. In total 12 students opted to be interviewed. All these students were between 19 and 22 years; six were seniors (who had studied for 1 year prior to COVID-19) and six were juniors who commenced their studies in June 2020. Three of the students had opted to return home and the rest had remained in the dormitory. The demographic details are provided in Table 1.

**Table 1:** Participants

No.	Pseudonym	Year of study	Gender
I	Poe Qwa	1st	Male
II	Kyaw Htun	2nd	Male
III	Poe Htoo	2nd	Male
IV	Kyaw Kyaw	2nd	Male
V	Saw Hla	2nd	Male
VI	Naw Snow	1st	Female
VII	Saw Kyaw	1st	Male
VIII	Naung Naung	1st	Male
IX	Paung Paung	2nd	Male
X	Naw Mu	2nd	Female
XI	Saw Lay	1st	Male
XII	Nay Lay	1st	Female

### Ethical approval

An ethical proposal was submitted to and approved by the University of Reading’s Institute of Education ethics committee. Along with standard issues, such as data protection, informed consent, and secure data storage, this project had particular focus on the use of insider research. In particular, care was given to ensure that no undue pressure was placed upon the students to participate and during the interview, the first author considered how to avoid using his insider knowledge to undermine the authentic narrative told by the participants (Floyd & Arthur, 2012). The initial proposal to conduct the research was discussed by the programme’s board of directors and approved before invitations were given to the students to participate. It was explained that this was extra-curricular and would not impact their academic progress. It is also intended that this study will form the basis of an introduction to research module for the students, as they are all intending to progress to university. Care was also given to the students’ anonymity. In addition to the use of pseudonyms, all identifiable place names and former school names were changed at the initial transcription stage and the specific age of each student has not been included in this paper.

### Data collection

We co-constructed the interview protocol, which was informed by the notion of resilience being a process in the literature review. Hence, the interview was semi-structured and organised chronologically into six sections covering the students’ previous educational experiences, their current experiences in Maung Kyaw Programme, their responses to both COVID-19 and political instability, their future plans, and an open section for further comments. Whilst the literature suggested several possible factors in the development of resilience, such as being valued by teachers, internal locus of control, and support of family members, the questions were carefully crafted so as not to lead the participants, but rather give the participants the freedom to select and articulate the experiences which they had found most relevant. For example, when exploring overcoming challenges, two initial questions were: “Did you ever want to stop your education? Why or why not?” and “When you were finding things difficult, what did you do?” Two students were interviewed in stage one, after which the interview protocol was revised with three additional questions added to the initial 14. Stage 2 involved the interviewing of the 10 remaining students. As the changes were minor, all 12 data sets were included in the analysis. The interviews lasted between 28 and 65 min.

### Data analysis

The interviews were conducted in English and transcribed removing verbal fillers and repetition as no discourse analysis was to be performed and this increased the readability of the data. Each interview was then thematically coded and iteratively analysed in the process outlined by Lichtman (2010), with the codes, categories and themes emerging from the analysis. An example of how codes were grouped into categories and an emerging theme is shown in Table 2.

**Table 2:** Example of coding for theme of community

Code	Category	Theme
Volunteered in quarantine centre	Examples of previous community service	Community service orientation
Helped to build school		
Responsible for others in quarantine		
Volunteered with community organisation		
Lead drawing class		
Saw others as brothers and sisters		
Saw need and fulfilled		
Volunteered as teacher		
Goal linked to helping community	Future plans to develop community	
Work in rural area		
Desire to make a difference		
Give free education, especially ethnic/rural		
Wants to bring peace to community		
Create job opportunities/open shop		
Desire is to serve and train		
Help those that don’t have same opportunity		
Improve health care		
Saw many school kids dropped out	Inspiration from community	
Community has low living standard		
Many left for jobs abroad		
Saw many unqualified teachers		
Many old teachers stopped due to low pay		

After the codes were refined, organised and grouped thematically, there were a total of 512 individual codes and 40 categories. No new categories were added after the 7th interview. However, new codes continued to be added due to the students describing unique experiences; the new codes for the last four interviews tended to be variations on existing codes. The decision was taken to keep the codes as close to the students’ own words as possible to give an emphasis to the students’ authentic voice; however, the drawback of this was the continued addition of new codes. The emergence of new codes and the cumulative total of codes and categories is summarised in Table 3.

**Table 3:** Code and category saturation

No.	New code	New code %	Cum. code %	New categories	New categories %	Cum. categories %
I	101	19.73	19.73	30	75.00	75.00
II	59	11.52	31.25	1	2.50	77.50
III	65	12.70	43.95	4	10.00	87.50
IV	51	9.96	53.91	2	5.00	92.50
V	56	10.94	64.84	1	2.50	95.00
VI	36	7.03	71.88	0	0.00	95.00
VII	65	12.70	84.57	2	5.00	100.00
VIII	21	4.10	88.67	0	0.00	100.00
IX	13	2.54	91.21	0	0.00	100.00
X	16	3.13	94.34	0	0.00	100.00
XI	14	2.73	97.07	0	0.00	100.00
XII	15	2.93	100.00	0	0.00	100.00
**Total**	**512**			**40**		

When looking for the saturation level, one key aspect to consider is the homogeneity of the sample. Guest et al. (2006), with a sample of 40 participants, found that the first six interviews were sufficient for revealing all the metathemes and first 12 for illuminating the themes. Hagaman and Wutich (2017) similarly found that less than 16 interviews were required to reach thematic saturation in a homogeneous sample. The target population of this study all had similar life experiences leading up to them studying in the same institute, during the 2019–2020 academic year, and thus the relatively small sample size of 12 was deemed sufficient to understand the lived experiences of the target population. No new categories were added after the 7th interview, suggesting thematic saturation had been achieved (Namey et al., 2016). Hence, we judge that this data set is a sufficient representation of the students in this institute.

## Findings

The iterative coding and thematic analysis revealed six major themes, of which five are pertinent to the subject of resilience. These were the ongoing process of resilience; goal orientation; emphasis on the community; basic needs fulfilment; and the impact of significant people. The sixth theme concerned the students’ general reflections on online learning and lays beyond the scope of this paper but would merit further research. The themes will be presented as they align to each of the three research questions. This section will focus on the data collected from the participants as it pertains to each of the three research questions; this will then be discussed in relation to the existing literature in the following section.

### RQ1: how did previous life experiences affect the students’ resilience?

Noticeable in all the interviews was the theme of how the resilience demonstrated was not a spontaneous response to the pandemic and subsequent nationwide instability, but rather the result of previous life experiences which demonstrated a pattern of overcoming challenges throughout their lives.

Significantly, none of the students came from affluent families. Four of the twelve, having fled civil war, were from refugee camps, four had family who worked for below the legal minimum wage, and seven had either parents or close family members who were subsistence farmers. Thus, they were all experienced in dealing with economic need. This is seen in Naw Mu’s reflection:

My parents divorce. My father and some of my siblings moved to refugee camp … My siblings who stayed with my mother didn’t have the chance to study because my mother couldn’t support them. … [Later] my father was not healthy, so two of my older brother, need to drop the school for work. So I’m the one that got the chance to keep studying. (Naw Mu, X)

In addition to personal hardship, the students’ educational experiences at a young age had often been difficult. One remembered moving to a government middle school, and learning in Burmese for the first time (he’d previously learnt in his native Karen language):

In school time, we were often beat. When I first started my studying in the local town, I didn’t understand Burmese language too much, so when my teachers gave the lesson, we have to memorise all the passage, all the words. But I don’t understand it, how am I going to memorise it? It is difficult for me. And my teacher beat me so often. (Nay Lay, XII)

As well as communication barriers and strict discipline, three students described failing the Myanmar government exam. For example:

In 2017 I failed my matriculation class exam. So, I thought that was the end of my education. But one of my friends, named Saw Taw, explained about GED. So, I have some opportunity to keep going on my education. (Poe Qwa, I)

Failing the Matriculation (end of high school) exam is a common occurrence in Myanmar, with only between 30 and 35% passing annually. This leads to many students dropping out of school, and others repeating, sometimes for several years. Like Poe Htoo and Naw Snow, after failing his exam, Poe Qwa had to choose whether to cease his education or go forward. With tuition and education being very expensive, he opted to move to Thailand, where a friend helped him into the migrant learning system and he eventually succeeded in entering a GED school. Moreover, whilst waiting for school to open, he taught himself programming languages using online resources, due to his “goal to keep on study[ing]”.

All 12 of the participants for this study went through prolonged periods of separation from their families to study, with three of the students leaving home before the end of primary school. Several of the students recounted the emotional impact this had had on them. For example, Poe Htoo stated about his time in high school:

So I didn’t contact my parents for two years. … sometimes I miss my family so much, I feel, really sad, and think about my family, I miss them, I don’t know how they are still doing, and sometimes I was crying alone. So, it is difficult for me to stay so far away from my family, … but I keep trying, because my purpose is to study and one day, I want to help my community. (Poe Htoo, III)

For Saw Kyaw he reflected on his nervousness for the future, and Nay Lay discussed how she even felt nervous when returning home after several years apart from her parents and being shy around them as it was no longer a familiar setting. Whilst challenging, these experiences also enabled the students to learn coping mechanisms from a young age. These took a variety of forms:

When I was sad, sometimes I try to pray, and sometimes I just listen to music, gospel song. This can encourage me, the word of the song, and the word of God, like that. So I feel better, better. Sometimes, I crying and I just fall asleep, and after I wake up it make me feel better. (Poe Htoo, III)

I read book that inspire people and give motivation for people … a book that gives people many inspiration. I like to read book in that time when I feel like that. (Naw Mu, X)

Teacher May always send me some posts that encourage people. Also some prayer and worship. Like this, also when we are texting, she always tell me things. (Naw Snow, VI)

For some, they drew on their spiritual faith (some were Christian, others Buddhist) to find the motivation to continue, others read about inspirational figures from history. Many of the students discussed listening to music, singing and other hobbies which also took their minds off their problems. Whilst the form of coping differed, common across the sample was that each had found a way to motivate themselves in the past whilst experiencing emotional, economic, or educational challenges. It was also noticeable that some deployed these mechanisms by themselves, and others were impacted by significant others. For example, in the quotes above, Poe Htoo expressed that he listened to the word of God, whereas Naw Snow explained that one of her teachers had shared the spiritual encouragement to her.

### RQ2: what internal/individual factors assisted students to be resilient in their studies?

Turning to the students’ reflections on the past year, one of the most striking themes from the data was the students’ focus on their goals, and how this drives them to make choices which would enable them to fulfil them. This was evident in their recollections of their thought processes even before they joined Maung Kyaw Programme. Commenting on why he chose this institution, Poe Qwa stated:

I have … two reasons. Maung Kyaw is two year GED programme, so I can have enough time to study, then 75% of students go to university, so I also have opportunity to go, maybe Rangsit [University]. That’s why I choose to study. (Poe Qwa, I).

The sentiments of Poe Qwa reflect those of the entire sample, who clearly showed they had made specific plans for their futures and were in the process of gaining the knowledge and skills needed to progress. Thus, when COVID-19 and the political upheaval occurred, the students were already invested in their visions for their lives and had both the positive drive to continue their studies, and the negative reinforcement of potentially losing their opportunities:

Without education, I lost my way, because only education can help me. Also some of my dreams, I want to help the people in our village, in our society, I want to go back and to help them, so if I am educated person … I hope I can help them. (Naw Snow, VI)

I believe I can’t stop my study. If I stop my study, I lose my hope, I lose everything. So I keep studying. (Nay Lay, XII)

These sentiments are again representative of the sample, with the variation occurring in the specific nature of their plans. Whereas some aim to become educators, others would like to work in health or become businessmen; notably, most expressed that similar notion of losing everything if they stopped their education at this stage.

More positively, a couple also shared how the difficult events of 2020 had enabled them to seek new opportunities. Naung Naung, for example, stated that he had “spare time to study other online subjects” and had sought out online programming courses to help him prepare for an ICT major at university. Significantly, 8 of the 12 participants stated that they felt better prepared for university, all but two stated they had learnt new skills for the future, and half felt they had become more independent and were, therefore, ready to study at university.

The second theme, closely linked to goal orientation, was the students’ focus on their communities. Many had previously volunteered in their communities, such as helping in a quarantine centre during the COVID-19 pandemic, and they were unanimous in expressly linking their goals to developing their communities.

I want to become nurse because I want to help my community. Like, especially in Karen rural area, there are a lot of people who, who have no access to a higher health care. So I want to help them., This my dream, since I was born. (Kyaw Htun, II)

I want to go to university to achieve my dream. I want to become a teacher, and I want to help my Karen people who have to flee and who have no opportunity to a high standard school, like other people or other citizens. (Saw Hla, V)

For me, I want to be a businessman for my future, because in my village, there are few jobs and most of teenagers, when they can read and write, they drop out of school and go to Thailand to find a job. So, I think if I become a businessman … it will create some jobs for them, so they will get some money and can go to school. (Kyaw Kyaw, IV).

Whilst it is natural for both Kyaw Htun and Saw Hla to link careers in nursing and education with serving their communities, the surprising response was from Kyaw Kyaw who saw a business major as a route towards creating job opportunities and developing communities. Thus, throughout the educational disruptions of 2020, the cohort not only had their own personal motivation to study to achieve their “dreams” for themselves, but also a burden of responsibility to become educated to improve the living conditions of their villages and communities.

### RQ3: what external/community factors assisted students to be resilient in their studies?

As well as the internal drive to succeed, two themes were revealed that linked to external factors encouraging the students to continue their studies. The first of these was their basic needs being satisfied. With gatherings limited, travel prohibited, and political upheaval, the most fundamental need that the students had was to be safe. Those who lived in nearby areas were able to return home and continue online, but those who had come from refugee camps, conflict areas or areas without access to the Internet were given the chance to remain on campus and study remotely from their dormitories. One commented:

This year was so difficult for us, not only for us, but so many people in the world. So as being a student when a school is close, … here we are safe because we don’t go outside too much, so we can keep studying safely. (Poe Qwa, I)

Beyond feeling secure, a second basic need was access to technology: devices to stream videos and do quizzes and an Internet connection which was stable. Whilst there were periods when the Internet was blocked nationwide, when there was Internet, students in the dormitory shared devices, and often allocated most of their pocket money for mobile data.

I also worry about the money because the online cost a lot of money. My parents also have problem with that … so I just save my money and just use my money just for studying, so I cannot use for other. I really have to manage that, I really have to manage my money carefully, because of online study. (Poe Htoo, III)

Clearly this links to the next theme under external factors: the impact of significant people, namely, family, friends, and teachers. Families generally had a beneficial impact on the students’ resilience in the face of adversity. Whilst only two of the students stated their parents had ‘no interest’ or knowledge about further studying options, eight explicitly stated their parents or siblings encouraged them to continue their studies. For example:

The time is difficult and I am the one who got the chance to continue my education, so they really want me to achieve my goal and they really want my education to be better than my siblings, so they really support me and tell me all the time, don’t give up. (Naw Mu, X)

In addition to family, community members were often a source of encouragement, with six stating it was a member of the community that had found the pathway for them to continue their studies. Once in school, friends became an emotional support too. Kyaw Htun, Kyaw Kyaw and Naw Mu all referred to the school community as a ‘family’ and Naw Snow stated.

Especially Ma Ma, she help me a lot. I feel I lost my way, … Not lost my way, but feeling I can’t do anything like this, so she encourage me. (Naw Snow, VI)

Friends also played an important role in online learning:

We comment on each other because, as we are different person, we have different opinion. Some people will have a lot of skill about the video presentation, the video editing … but for some people, maybe it is new for them … and that’s why we comment on each other. It doesn’t mean that we are better than each other, because we have to help each other and make sure for the next time. (Saw Kyaw, VII)

Finally, during the pandemic and political crisis, the teachers were also crucial as they worked to find solutions to the ever-changing situation such as moving to Moodle from Facebook when access became restricted and distributing work on USBs when the Internet access was cut. The students commented on both the educational and emotional support:

The feedback is very encourage and important for me, like a mirror, what you need to do to prepare so we can know this, so very encourage for us. (Naung Naung, VIII)

This was hard year … you may have depress and stress, so when you feel that kind of thing, teacher can meet us and encourage us to keep strong, and study hard. (Kyaw Htun, II)

## Discussion

At the outset, the first research question aimed to explore the link between previous life experiences and expressions of resilience. Our findings show that all the participants demonstrated a pattern of overcoming a variety of adverse situations including economic disadvantage, family instability and national conflict. Each developed a variety of coping mechanisms including self-reflection, support from family members and educational figures, and spirituality, which demonstrates the advantage of exploring this topic through the socio-ecological framework of Bronfenbrenner (1979). These sources of support are consistent with the existing literature (see Dass-Brailsford, 2005; Motsa & Morojele, 2017).

Concurring with findings from Smokowski et al. (1999), this paper demonstrated that the formation of resilience is an ongoing process. Whilst each of the students’ journeys were different, common adversities faced included separation from parents at a young age, a lack of financial resources to attend formal schools, and frequent relocation in both their homeland and across the border in Thailand. Each of these adverse situations caused the students to build resilience, and significantly this study shows that once effective models of resistance have been adopted, they can be applied to the sudden onset of a new adverse situation. In terms of this study, the participants were able to utilise their resilience to first overcome the challenge of studying during COVID-19 restrictions and then to persevere in education whilst living in a nation struggling with political and economic instability. Notably, having previously succeeded in utilising prayer, support of family members and the encouragement of teachers to overcome hardships, these responses were deployed in response to the challenges of the last academic year as a means of enabling the students to progress through the adversity encountered.

This study has theoretical implications as it adds to the growing literature demonstrating how students from lower socio-economic backgrounds can succeed in education. In the context of this study, refugees who fled their homeland and children of below minimum wage workers, were successful when models of resilience were fostered, and demonstrated their ability to thrive through preparation for university. At the time of publication, seven have begun their university studies and the remaining five have been accepted and will presently be commencing their studies. This ability to thrive is consistent with research into students from lower socioecological status in the United States, Africa, and Asia (Arnold & Doctoroff, 2003; Henderson et al., 2016; Sandoval-Hernándex & Bialowski, 2016).

At the centre of Bronfenbrenner’s ecological framework is consideration of the individual. In terms of these participants, a common view was that education was a gateway to a brighter future, which also reflects the findings of Motsa and Morojele (2017) in Swaziland and Rogers and Anderson (2019) in Cambodia. Of all the themes identified, the emphasis on goal fulfilment was the most universally expressed, and the proximity of the participants to applying to university further enhanced their drive to succeed. The exploration of the thick data descriptions of the students’ aspirations enables this study to move beyond the causality established by Wills and Hofmeyr (2019) and determine in this population, one of the causes of resilience formation was the students’ focus upon progressing towards their goals.

Yet it was striking that all the participants of this study had a strong sense of belonging to their local community and a desire to serve, where they could in their home region, thus they articulated that the goal of their educational pursuits was for the benefit of their people. This can be seen in the examples of Kyaw Kyaw who linked his goal of studying business, to the ambition of creating jobs for his community, and Kyaw Htun who explained his determination to study nursing in terms of an acute need for increased access to medical care in his rural community. As in Motsa and Morojele (2017), the similar findings regarding goal orientation and community development increasing motivation suggests potential for those in disadvantaged communities to become more resilient when the goals of the microsystem and mesosystem are aligned.

Looking at the mesosystems, the students expressed that they had been influenced by a variety of different external actors. For some, it was their parents who gave them the encouragement to study, for others their parents were uneducated and offered little or no encouragement to further their studies. Other significant figures included teachers, and community members who gave advice and helped the students transition between institutes. All of them mentioned at least one adult who guided them and acted as a role model, which supports the assertion of Arnold and Doctoroff (2003) that older figures act in a protective way throughout the resilience formation. This is also revealed through some of the students’ references to prayer and spirituality. The students in this study were a mix of Buddhists and Christians. Though not specifically asked about religion, as the goal was to allow the students to articulate the factors that they felt were most significant, three of the students described the way they relied on their spiritual faith in times of need. This aspect cuts across the levels of Bronfenbrenner’s model, as at the exosystem level is the culture of religion, which is modelled in practical ways by significant adults at the mesosystem level and then internalised and used by the individual.

One final aspect that was particularly relevant to this study, which is less explored in resilience studies due to many being conducted in western contexts, is the fundamental requirement for certain needs to be fulfilled. At the individual level, the participants needed access to a computer and money for mobile phone data. At the mesosystem level, this involved negotiating with parents, siblings, and teachers to ensure they had sufficient resources. At the wider exosystem level, the termination of Internet services during the political instability affected them, as did the need for safety. As with Landis et al. (2018), the educational campus, which was available throughout to those unable to travel, was perceived as a safe space. Whilst resilience helped the students to overcome the daily instability, these fundamental needs all needed to be fulfilled before effective learning could occur. This demonstrates that satisfaction of Maslow’s hierarchy of needs is an essential requirement for forming the exosystem in which resilience can be demonstrated.

## Conclusions

In summary, this paper explored the presence of resilience amongst a group of students in Myanmar, who had successfully studied online throughout the spread of COVID-19, and widespread political instability. It was evident that the success the students demonstrated in deploying resilience strategies was the result of many lived experiences which formed a pattern of resilient actions. This resilience was driven internally by the students’ desire to fulfil their goals and serve their people, and externally through the provision of basic needs and through the support and encouragement of family members, friends, and teachers.

One limitation is the size and scale of the study, with all 12 participants coming from a single programme and all the participants being students. A wider study including students from other programmes may offer different results, as would a study which included both students and teachers as participants. Moreover, the purposive sampling criteria adopted excluded those who failed to be resilient, and it would be revealing to conduct a similar study with those who failed to adapt to see whether some of the factors identified here were missing, or if there are other aspects not revealed by focusing on those who succeed.

In addition, it is true that a qualitative study of this kind cannot draw universal conclusions regarding resilience formation. However, the consistency of these findings with studies conducted elsewhere, along with the transparency regarding decisions taken, helps to build credibility in the conclusions drawn. Moreover, one of the strengths of this paper is the unique view it gives into an under-researched educational context which has struggled and adapted throughout decades of civil war, under-investment, and more recently, a health pandemic and political change.

The five traits of resilience demonstrated by all the participants in this study were focusing on goals, serving the community, drawing on past experiences, involvement of significant others, and fulfilment of basic needs. One of the key theoretical and practical implications is that as students can develop the capacity to demonstrate resilience, educators should incorporate activities which seek to develop these steps to success throughout the students’ educational journeys so that when adversity occurs, the students will be better able to adapt and thrive. Whilst the delivery of resilience training is growing internationally, developing countries such as Myanmar face both a lack of basic educational facilities and ongoing under investment in the education sector, leaving few resources for non-core activities. However, with limited in-service training, teachers could be encouraged to promote resilience building through their lessons, thereby assisting students develop coping mechanisms, akin to those developed naturally in students who have experienced adversity, in a simple, low-cost approach. To achieve this, our findings suggest that teachers should build into their lessons opportunities for problem solving skills and coping strategies to develop. Through mentoring and personal development classes, teachers can assist students to reflect on their past experiences and plan how to achieve their future goals whilst simultaneously ensuring that they feel supported emotionally and have the key equipment or space to be able to engage successfully with their educational endeavours.

Future research would benefit from expanding the scope of this study to explore the experiences of students in other educational programmes, both in Myanmar and other low-resource settings. Future research could also interview a wider range of stake holders such as teachers and administrators to give a broader perspective. Moreover, whilst this study explored how resilience had been used to facilitate the successful completion of the academic year during COVID-19, the resilience definition we adopted explicitly stated that resilience involves bouncing back and thriving. Whilst all the students commented on feeling well prepared for commencing university, at the time of writing this is merely their perception. A follow-up study is currently underway to track some of these students’ experiences in starting university and comparing them to students in previous cohorts who started university with no previous online learning experience.

Finally, there are implications for future practice, which need to be researched further. The data analysis revealed a sixth theme relating to benefits of online education, which fell outside the scope of this paper. Future research, complimentary to the work underway in the UK by Open University and in Italy by Appolloni et al. (2021), is, therefore, planned to explore how advances in the use of ICT during COVID-19 can be integrated into the curriculum in low-resource contexts once students return to the physical classroom.

## Supplementary Information

Below is the link to the electronic supplementary material.

Supplementary file1 (DOCX 15 kb)

## Footnotes

1 It is beyond the scope of this paper to address these issues in more detail. Moreover, conducting research in such a setting means it is an ethical imperative to focus on the safety of all involved. Thus, readers are advised to search elsewhere for a more in-depth look at the nationwide situation in Myanmar following events of February 1, 2021.
