# Supplementary material for: Resilience: Myanmar students’ experiences of overcoming eLearning challenges during COVID 19 and political instability
Source: Asia Pac Educ Rev. 2022 Aug 15:1–13. Online ahead of print. doi: 10.1007/s12564-022-09781-6 (PMC9376570; doi:10.1007/s12564-022-09781-6)
Supplement: Supplementary file 1 — Supplementary file1 (DOCX 15 kb) [file 12564_2022_9781_MOESM1_ESM.docx]

**Appendix: Revised Interview Schedule After Stage 1**

| **Introduction, explain purpose of research and confirm ethical approval** | |
| --- | --- |
| **Section 1: Background and life experiences** | **1. Can you tell me a little about your family background?**  **2. Can you describe your educational journey?**   - **Why did you apply to Brighter Futures?** - **What is your future career goal?** - **How would you describe your experience on the programme?** |
| **Section 2: The Education Delivered During 2020-21** | **Please think back over the year.**  **3. How was education delivered at the start of the school year?**   - **What happened then?** - **How did the style of education change?** - **How did you get feedback from your teachers?**   **4. How did your hobbies change during the year?**  **5. How did Covid-19 affect your lifestyle?**  **6. Did you know anyone affected by Covid e.g. who had to quarantine?**   - **How did this impact on your educational experiences?**   **7. How did things happening in Myanmar affect your desire to study?** |
| **Section 3: The Participant’s Previous Exposure to ICT** | **8. Before you came to Brighter Futures, how would you describe your level of computer skills?**   - **How often did you use a computer?** - **How often did you use a phone?** - **How had you used a computer or phone for your education?** |
| **Section 4: Reflecting on the Education** | **9. You said you studied by [style of education]. What did you enjoy about this?**   - **What were the difficulties that you found?** - **How did you try to solve these problems?** - **How did your classmates help you?** - **How did your teachers help you?** - **Did your parents or others in your community discuss your education with you? If so, what did they say?** - **What opportunities arose from this experience?** |
| **Section 5: Reflecting on keeping going** | **10. Did you ever want to stop your education? Why or why not?**  **11. When you were finding things difficult, what did you do?**   - **What helped you keep going with your education?** |
| **Section 6: Wrapping up** | **12. Do you think you benefited from this style of education? How or why not?**  **13. Is there anything else you would like to say about the education over the past year?**   - **When Covid is over, what, if any, of the changes to education should be retained?**   **14. What are your plans for further studies?**   - **How might this experience impact on these?** |
